# Apoplexy

**Published:** 1889-08

**Authors:** 


### APOPLEXY.

A blood vessel of the brain has lost some of its elastic strength ; food is abundant ; digestion is good ; blood is made in abundance, but little is worked off by exercise ; the tension on every artery and vein is at a maximum rate ; the even circuitous flow is temporarily impeded at some point throwing a dangerous pressure on another ; the vessel which has lost its elastic strength gives way, blood is poured out, a clot is formed which, by its pressure on the brain, produces complete unconsciousness, This is the apopletic stroke. It will be perceived that there are two leading conditions upon which the production of the stroke depends ; a lessoned strength in the vessel, and an increased tension on it.

